# Longitudinal analysis of geographical inequalities in retail density of tobacco and nicotine vapour products in Scotland, 2020–2024

**DOI:** 10.1016/j.pmedr.2026.103611

**Published:** 2026-08-25

**Authors:** Chunyu Zheng, Niamh Shortt, Asiya Hamid, Andy MacGregor, Amber-Jane Morgan, Allison Ford, Jamie Pearce

**Affiliations:** a Centre for Research on Environment, Society and Health (CRESH), School of GeoSciences, University of Edinburgh, Edinburgh, UK; b Scottish Centre for Social Research (ScotCen), Edinburgh, UK; c Institute for Social Marketing and Health, University of Stirling, Stirling, UK

**Keywords:** Commercial determinants of health, Neighbourhood retail environment, Availability, New nicotine products, Tobacco, Socio-spatial inequalities, Longitudinal analysis

## Abstract

**Objective:**

Legislative efforts have aimed to reduce the visibility and accessibility of tobacco to limit use and prevent uptake, but less attention has been paid to emerging new nicotine products (NNPs). This study examines changes in the density and socio-spatial distribution of tobacco and nicotine vapour products (NVPs, the primary type of NNPs) in Scotland between 2020 and 24.

**Methods:**

National register data from 2020 to 2024 were used to examine changes in the count and density of outlets selling tobacco and/or NVPs across Scotland. Kernel Density Estimation (KDE) was utilised to assess outlet density in neighbourhoods, differentiated by income deprivation and urban-rural categories. Trends were analysed using non-parametric methods and stratified by outlet types.

**Results:**

Over the study period, the number and density of registered outlets remained constant overall, with slight increases in outlets selling tobacco and NVPs, outlets selling NVP-only, and declines in tobacco-only outlets. Income deprived neighbourhoods and large urban areas had a persistently higher density of outlets selling tobacco and NVPs, with socio-spatial inequalities widening continuously.

**Conclusions:**

The study underscores the necessity for comprehensive regulatory approaches to reduce the availability of tobacco products and NNPs to minimise public health risks and address inequalities in exposure among vulnerable populations.

## Highlights

• Little legislative efforts have been paid to new nicotine products such as vapes.
• There has been a rise in the provision of registered outlets selling vapes in Scotland.
• Greater provision has consistently been found in the most deprived and urban areas.
• The socio-spatial inequalities in the retailer provision have been widening over time.
• Effective regulations to reduce tobacco and vape availability are anticipated.

## 1 Introduction

There is increasing international interest in reducing the availability of unhealthy commodities, including tobacco, alcohol and ultra-processed food, as part of national strategies to address the prevalence of non-communicable diseases (NCDs) and reduce health inequalities. For example, restricting the retail availability of tobacco products is recommended as key component of a tobacco endgame strategy (Moon et al., 2018; Kong and Henriksen, 2022). In the UK, the four devolved administrations have ambitious targets to become ‘smokefree’ (whereby adult smoking prevalence falls to 5% or less) over the next 5–7 years (Department of Health and Social Care, 2023). Recent legislation across many jurisdictions has notably diminished the visibility and appeal of tobacco products, particularly among adolescents, and has contributed to the denormalization of smoking behaviours (Kuipers et al., 2020; He et al., 2018). In Scotland, research indicates that such measures have decreased perceived accessibility and susceptibility to smoking in Scottish youth, thereby curbing initiation rates (Kuipers et al., 2020; Ford et al., 2020).

Despite these advancements, the emergence of New Nicotine Products (NNPs) such as vapes and nicotine pouches presents new challenges for public health, primarily due to the current regulatory gap concerning their retail and display (Al-Delaimy and Sim, 2021; Yan et al., 2023). The unrestricted retail availability of NNPs, coupled with a lack of stringent display regulations, raises concerns about the inadvertent promotion and accessibility of nicotine products to adolescents. This is important because the health hazards of nicotine and emerging NNPs remain unclear (Kaur et al., 2025). Further, there are concerns that NNPs may serve as a gateway to conventional tobacco use among younger populations (Golder et al., 2025), sparking debates about the necessity for an inclusive commercial tobacco endgame strategy that addresses both traditional cigarettes and emerging nicotine delivery systems (Bhatnagar et al., 2019; The Lancet Global H, 2024). Concerns are not solely focused on the individual health implications but extend to the broader public health impact, particularly regarding the potential normalization of nicotine use through these new products (Bhatnagar et al., 2019).

In the UK, there are currently no restrictions on where tobacco or NNPs can be sold. The Kahn review in England examined the steps needed to achieve the smokefree targets and recommended the introduction of a tobacco licence for retailers to regulate the number and location of where tobacco is sold, but this was not extended to NNPs (OBE JK, 2022). The review recognised that protecting children means restricting access to nicotine products but also acknowledging the need to balance this with supporting NNP use for adults attempting to quit smoking. Recent work in Scotland has shown that there is uneven distribution of tobacco retailing across regions of the country, with density much higher in more socially disadvantaged neighbourhoods (Shortt et al., 2015; Pearce et al., 2020). Further, a study using GPS data to calculate individual-level activity spaces of 692 children across Scotland found that children from the most disadvantaged neighbourhoods were exposed to seven times the frequency of tobacco retailing than children from the least deprived neighbourhoods (Caryl et al., 2019). Other studies in Scotland have consistently demonstrated a connection between the neighbourhood density of tobacco retailing and smoking behaviours among children (Shortt et al., 2016; Tunstall et al., 2018) and adults (Pearce et al., 2016; Clemens et al., 2020).

Whilst international work on geographical inequalities in the availability of tobacco retailers is well developed (Kong et al., 2025), few studies have examined the availability of NNPs and their socio-spatial distributions. Cigarette smoking has been the most common form of nicotine consumption, however, the use of NNPs is increasing among adolescents and young people (Brose et al., 2025; Hammond and Reid, 2025; MacGregor et al., 2025; Jackson et al., 2026). Recent research has suggested that vaping has already overtaken smoking in Britain in 2024 (Iacobucci, 2025). The lack of research on the availability of NNPs is a key barrier to developing evidence-based policies concerning their retail display and promotion, which further hinders the development of evidence-based policies aimed at safeguarding youth from premature nicotine addiction and its consequent potential health repercussions. Given this increased use of NNPs, and as part of a wider study investigating the retail availability, display, and marketing of NNPs in Scotland (NIPS study), this study aims to examine the longitudinal trends in the density of retail outlets selling tobacco and/or nicotine vapour products (NVPs, the most prevalent type of NNPs). We address two research questions: (a) how has the total count and density of tobacco and NVPs changed in recent years (2020–24) in Scotland and (b) how the relationship between retail outlet density and neighbourhood measures of deprivation and urban/rural categories have evolved.

## 2 Methods

### 2.1 Study design and population

The Tobacco and Primary Medical Services (Scotland) Act 2010 requires the registration of all retailers selling tobacco products. From April 2017, the tobacco products register was expanded to include NVPs (The Scottish Government, 2025). Annual extracts (collected between October–December 2020–2024) of the National Register were collated after removing duplicate records. All included registered outlets were geocoded using the postcode point data derived from the Scottish Postcode Directory (2025 v1). Field audits were conducted among 88 registered outlets derived from the March 2025 extract in four school catchment communities of Scotland between October and November 2025. The register data is largely consistent with audit results. Apart from 4 outlets missing from the register, 95.2% of the listed outlets were matched to the audited results based on address. Among these matched outlets, 87.5% had the same outlet type as recorded in the audit.

### 2.2 Measures

#### 2.2.1 Density measure

Kernel Density Estimation (KDE) was used to measure the density of registered outlets. By transforming the geolocations of outlets into a continuous ‘surface’ that divided Scotland into 50 × 50 m grid cells without any constraints by administrative boundaries (Shortt et al., 2015), KDE estimates the number and proximities of outlets of each cell within a given search radius (i.e., 800 m in our case, an approximate 10-min walking distance), through which greater weights were assigned to outlets that were centred and less to those further away (Shortt et al., 2015; Pearce et al., 2020). Therefore, the KDE values represent the proximity-weighted density of outlets per km^2^. Neighbourhood-level density was calculated from the KDE value of the cell at the population-weighted centroid of the datazone (500–1000 residents, akin to a neighbourhood) which is an estimate of where the majority of the population resides. The KDE measures were created across outlet types (defined by the products being sold) in each year (2020–2024). We firstly introduced three types of outlets provided by the National Register, which were: 1) outlets selling tobacco products only (‘tobacco-only outlets’); 2) outlets selling NVPs only (‘NVP-only outlets); 3) outlets selling both tobacco and NVPs concurrently (‘outlets selling tobacco and NVPs'). Further, three extra aggregate categories were created, which were 4) the combination of categories 1-3 (‘all’); 5) the combination of categories 2 and 3 (‘outlets selling NVPs'), to provide an overview regarding the longitudinal changes for selling NVPs; and 6) the combination of categories 1 and 3 (‘outlets selling tobacco’, to provide an ‘all tobacco’ category that is a more comparable measure with the previous study) (Pearce et al., 2020).

#### 2.2.2 Area-level attributes

The Income Domain of the Scottish Index of Deprivation (SIMD) 2020 was used to identify neighbourhood-level deprivation (Q1: most deprived, Q5: the least deprived). It uses the proportion of the neighbourhoods' population in receipt of means-tested state benefits. Neighbourhood urban-rural categories were based on the 6-fold urban-rural classification (2020) provided by the Scottish Government. These area-level measures were linked to the corresponding datazone in which the registered outlets were located (2011 geographies).

### 2.3 Statistical analysis

Changes in national-level counts and neighbourhood-level densities of registered outlets were examined using descriptive statistics. Given the non-parametric distribution of neighbourhood-level density measured by KDE and the large number of neighbourhoods with zero densities (for NVP-only outlets) across Scotland, we divided all neighbourhoods into non-zero-density neighbourhoods and zero-density neighbourhoods. The median with bootstrapped 95% confidence intervals (CI) from 1000 resamples was used for describing the longitudinal changes of density in non-zero-density neighbourhoods. For zero-density neighbourhoods, we used the proportion of neighbourhoods with zero densities across Scotland to assess changes over time (Supplementary I). To capture the geographic variation over time, we further stratified the trends by income deprivation quintiles and urban-rural categories. All analyses were disaggregated by outlet types. For sensitivity, neighbourhood-level availability measured by the count per 1000 population per datazone (‘count measure’) and the count of outlets at the local authority level were calculated (Supplementary II and III, respectively). The KDE measure was created using ArcGIS Pro (version 3.5.2), while all the analyses were performed in R (version 4.4.1). R packages, including ‘Tidyverse’, ‘Boot’, and ‘ggplot2’ were used for data cleaning, calculating bootstrapped confidence intervals of the medians, and making descriptive plots.

### 2.4 Ethics

The ethics clearance was received from the School of GeoSciences Research Ethics & Integrity Committee, the University of Edinburgh (Ref: 2024–917).

## 3 Results

### 3.1 Count of registered outlets at the national level

**Fig. 1:**
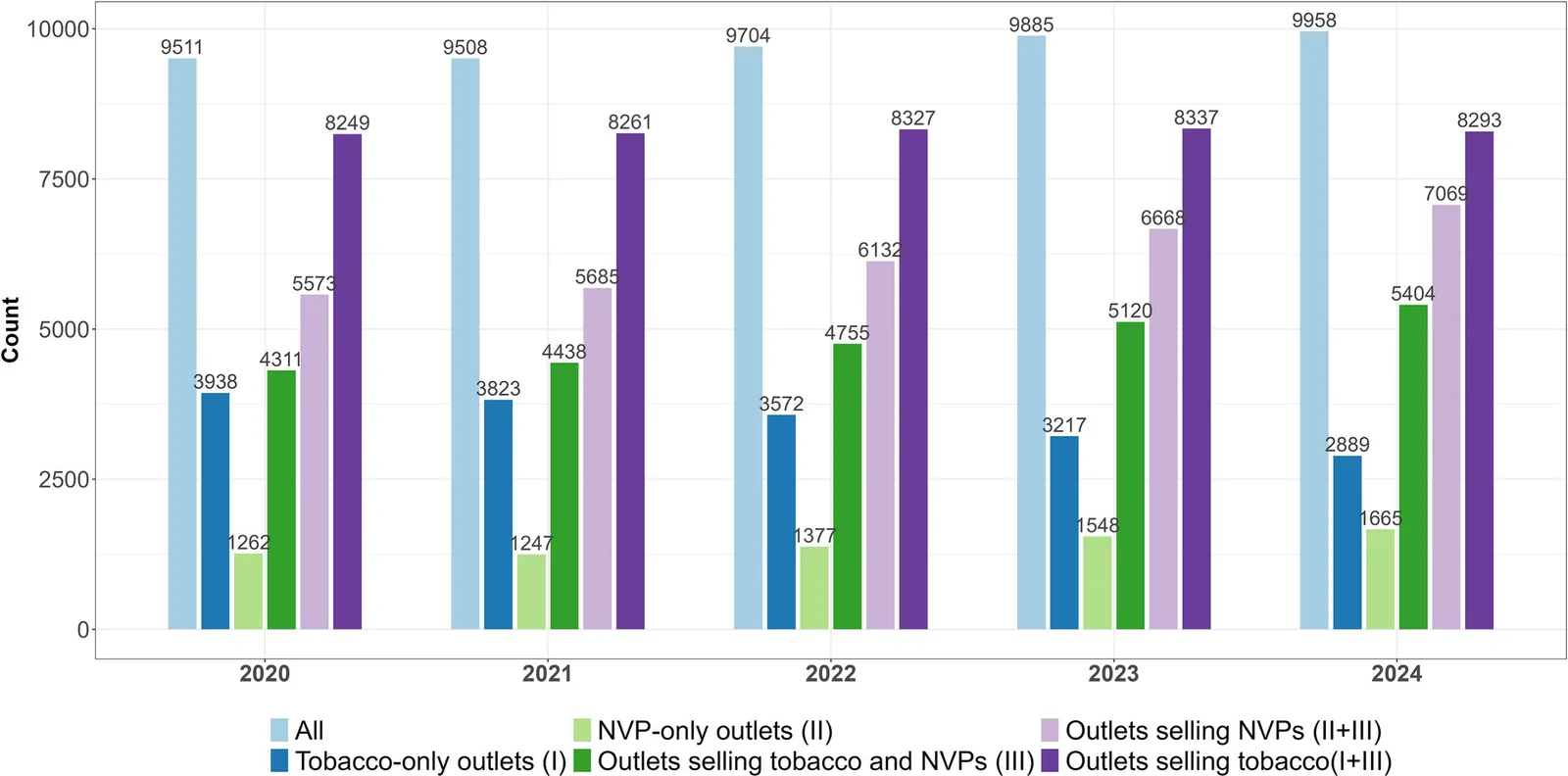
Total count of registered outlets at the national level in 2020–2024, by types of products being sold. NVP: Nicotine Vapour product(s).

The total number of registered outlets in Scotland increased from 9511 in 2020 to 9958 in 2024 (with a small decrease during the Covid period in 2021) (Fig. 1). Over this period, there was a rise in the total count of NVP-only outlets from 1262 to 1665. Substantial increases were found for outlets selling tobacco and NVPs (a 25.4% rise) and outlets selling NVPs (a 26.8% increase). Tobacco-only outlets were the only type showing a decrease, with a reduction from 3938 (2020) to 2889 (2024). Importantly, although the number of tobacco-only outlets has fallen over the study period, the total number of outlets selling tobacco showed a small increase from 8249 to 8293.

### 3.2 Density of registered outlets at the neighbourhood level

#### 3.2.1 Changes over time

**Fig. 2:**
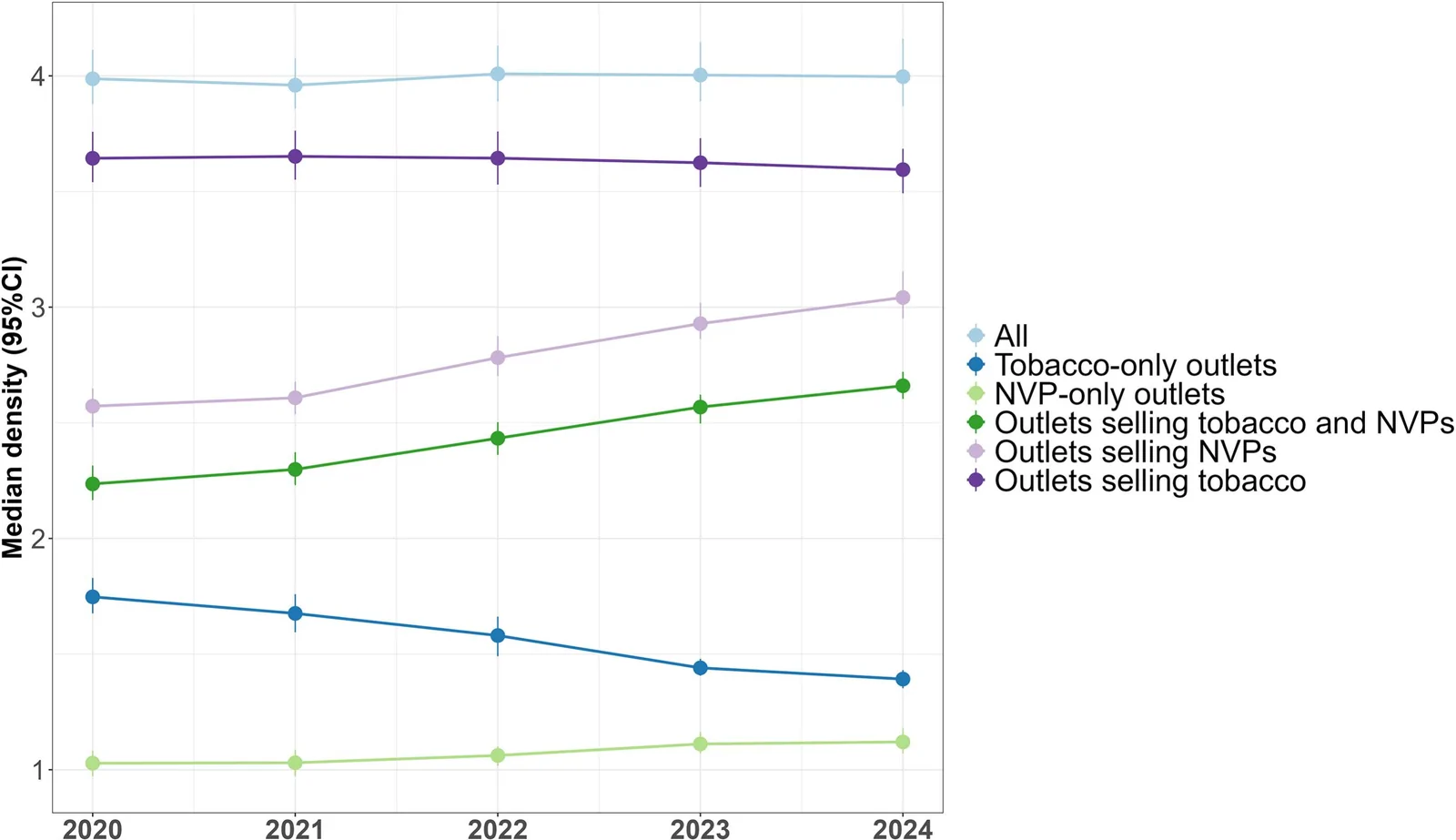
Changes in neighbourhood-level density (using KDE, 95% CI), 2020–2024, by product type. CI: Confidence Interval.

Similar to the trends in the count of outlets, the median neighbourhood-level density of all types of outlets remained mostly constant (around 4 outlets per km^2^) over time (Fig. 2). When the results were disaggregated by type, tobacco-only outlets demonstrated a decrease in the median density, from 1.75 (95%CI 1.67, 1.83) to 1.39 (95%CI 1.35, 1.43), while outlets selling tobacco remained mostly stable over time (from 3.64 [95%CI 3.53, 3.76] to 3.59 [95%CI 3.48, 3.69]). Concurrently, there was an upward trend observed for the other types of outlets. The median density of outlets selling tobacco and NVPs increased from 2.24 (95%CI 2.17, 2.31) to 2.66 (95%CI 2.60, 2.72). Similarly, the median density of outlets selling NVPs increased from 2.57 (95%CI 2.48, 2.64) to 3.04 (95%CI 2.96, 3.15) in 2020–24. The median density of NVP-only outlets was consistently lower than other types of outlets, but a gradual increase was noted from 2020 to 2024, from 1.03 (95%CI 0.97, 1.08) to 1.12 (95%CI 1.07, 1.18).

Consistent with the patterns described above, the proportion of neighbourhoods with zero densities remained stable over time for ‘all’ (around 10.9%) and ‘outlets selling tobacco’ (around 11%), with the only increasing trend noted for ‘tobacco-only outlets’ (from 27.1% in 2020 to 37.5% in 2024). The decreasing proportions were found for ‘NVP-only outlets’ (from 54% to 47.8%), ‘outlets selling tobacco and NVPs’ (from 14.8% to 13.2%), and for ‘outlets selling NVPs (from 14.3% to 12.8%) (S Fig. 1, Supplementary I).

#### 3.2.2 Changes by neighbourhood deprivation quintiles over time

**Fig. 3:**
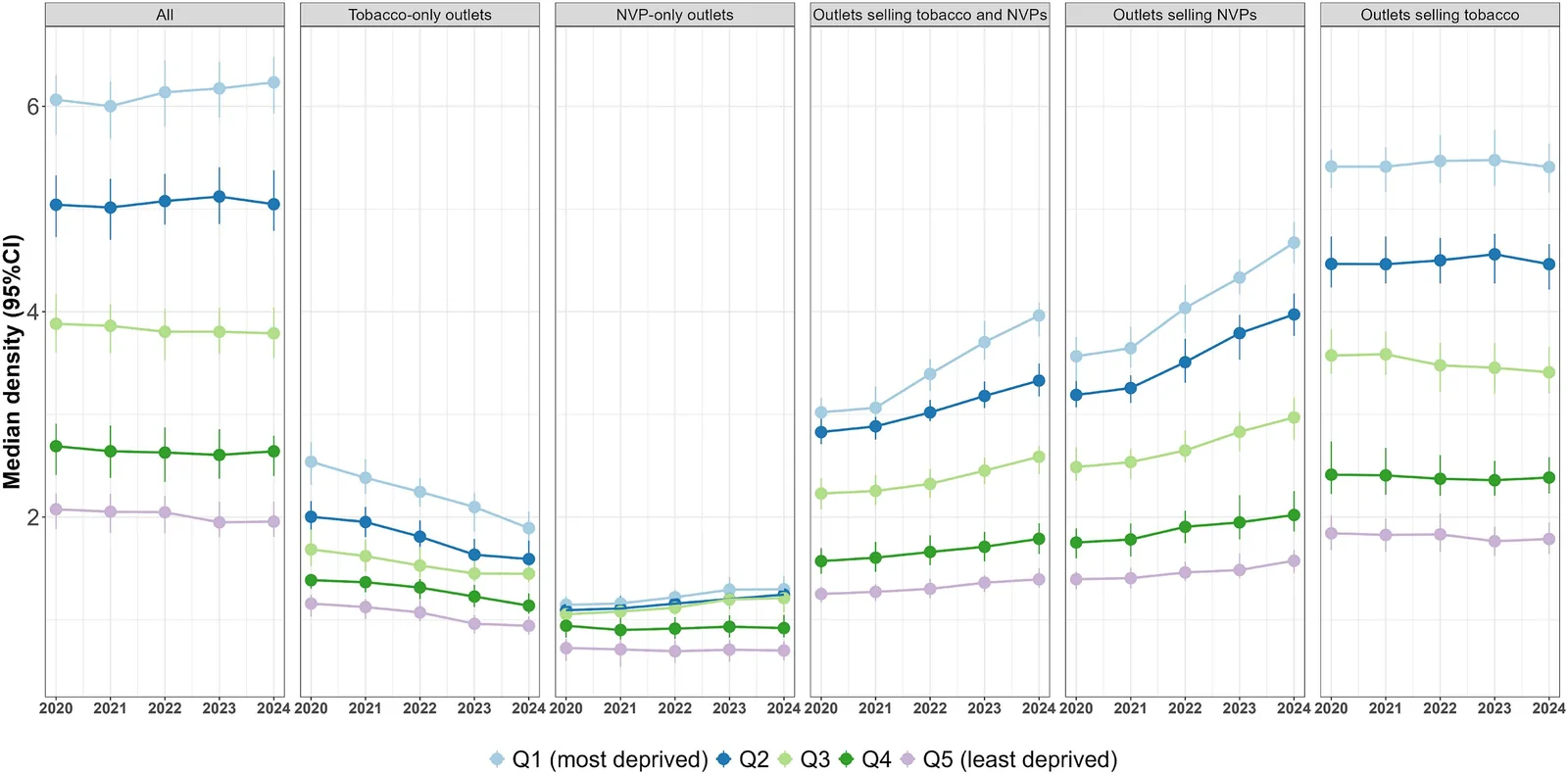
Changes in neighbourhood-level density by income deprivation quintiles, 2020–2024, by product type.

There is a clear social gradient in the neighbourhood-level density of registered outlets in all categories (Fig. 3). Over the study period, neighbourhoods with higher levels of deprivation had a consistently higher density of outlets, regardless of the types of outlets. When all-types of outlets were examined, there was a slight increase in the gradient over the study period, driven by both the greater increase in the most deprived quintile (from 6.06 [95%CI 5.70, 6.32] to 6.23 [95%CI 5.93, 6.49]) and a minor reduction in the least deprived quintile (from 2.08 [95%CI 1.89, 2.26] to 1.96 [95%CI 1.81, 2.14]). The widening deprivation gradient in density was also observed for NVP-only outlets and particularly pronounced for outlets selling tobacco and NVPs and outlets selling NVPs. For outlets selling tobacco and NVPs, the difference in the median density between Q1 and Q5 grew from 1.77 (2020) to 2.57 (2024) due to a sharper rise in more deprived neighbourhoods. Although there was a reduction in the median density of tobacco-only outlets across all quintiles, the most pronounced was found in the most deprived quintile. The median density of outlets selling tobacco showed a small decline in neighbourhoods with medium-level deprivation, but the social gradient remained mostly constant over time. Consistent patterns were observed for zero-density neighbourhoods (S Fig. 2, Supplementary I).

#### 3.2.3 Changes by urban-rural categories over time

**Fig. 4:**
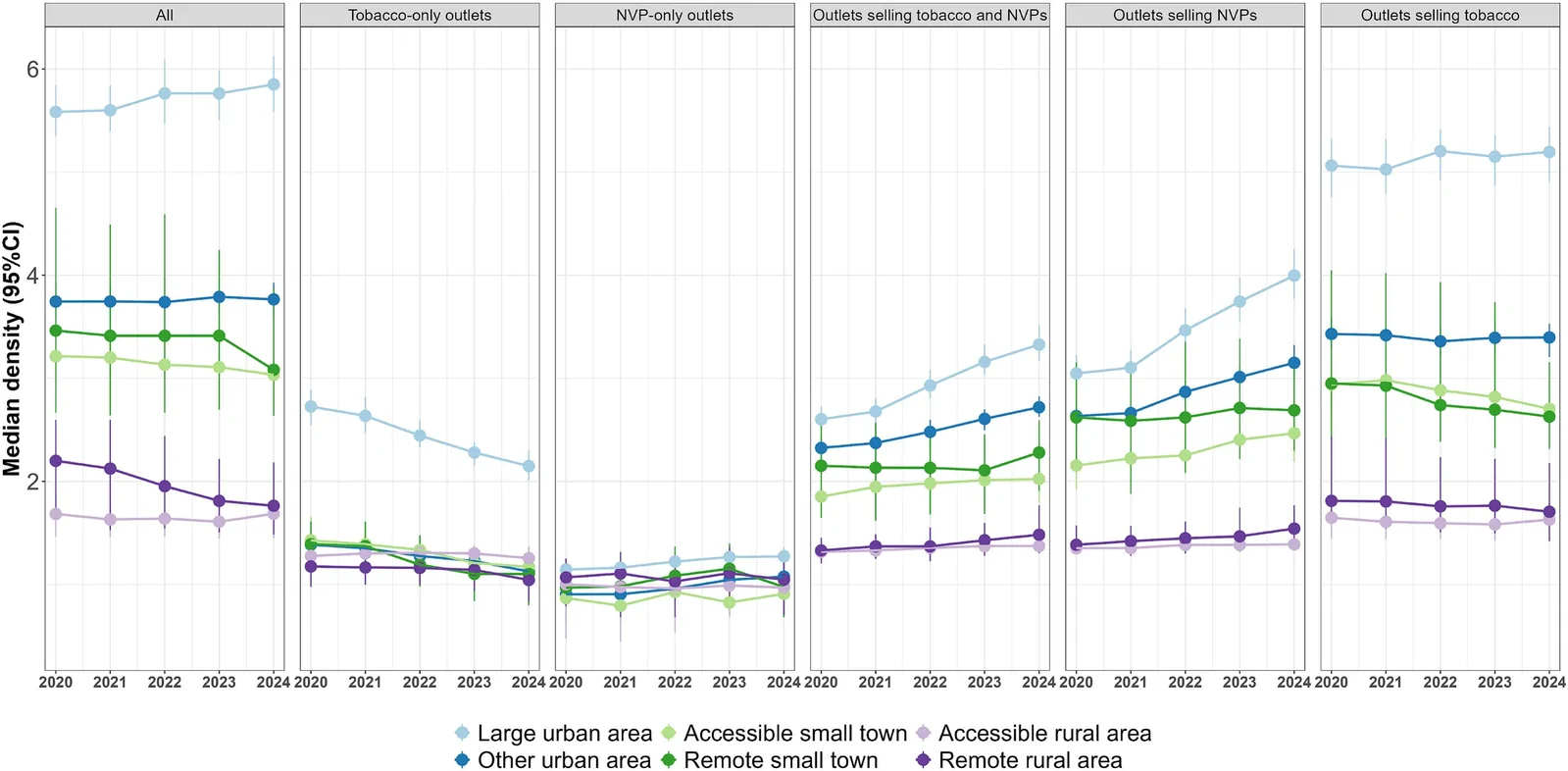
Changes in neighbourhood-level density by urban-rural categories, 2020–2024, by product type.

Examining the results by types of urban-rural categories, as expected, the median densities were consistently higher in large urban areas (Fig. 4). When all-types of outlets were examined, there was a clear gradient from large urban areas to rural settings, while within rural settings, the overall densities were higher in remote rural areas than in accessible rural areas. There was a slight increase in the median density across the study period in large urban areas (from 5.60 [95%CI 5.36, 5.88] in 2021 to 5.85 [95%CI 5.59, 6.13] in 2024) and a decrease or no change across the remaining urban/rural categories. A similar pattern was observed for outlets selling tobacco, however, there was no dramatic declining trend in remote rural areas. For outlets selling tobacco and NVPs /outlets selling NVPs, there was an overall increase in the median density across all urban/rural categories, with greater increase noted in more urban areas. Tobacco-only categories demonstrated a different trend, with a decrease in the median density across urban-rural categories, particularly in large urban areas where the median density fell from 2.73 (95%CI 2.55, 2.89) to 2.15 (95%CI 1.98, 2.30). However, little difference in densities of NVP-only outlets across types of urban-rural categories was captured over time. Patterns for neighbourhoods with zero densities, aligned with those observed for non-zero-density neighbourhoods, were presented in S Fig. 3, Supplementary I.

### 3.3 Sensitivity test

The analyses were repeated using the count of outlets per 1000 population per neighbourhood to test the sensitivity of the findings to the choice of density measure (KDE). The results were broadly consistent with those obtained using the KDE measure, though differences were observed, the overall substantive findings were unaffected.

## 4 Discussion

This national-level analysis shows that the number and density of registered outlets selling NVPs in Scotland have increased gradually between 2020 and 2024. Meanwhile, the total number of outlets selling tobacco products showed a small increase, although this was accompanied by a decline in the number and density of tobacco-only outlets with a pivot towards outlets selling both tobacco and NVP products. The findings also demonstrate clear and consistent socio-spatial differences in neighbourhood-level retail density for all types of outlets, apart from NVP-only outlets, with greater retailer provision in the most deprived and large urban neighbourhoods.

Earlier longitudinal work in Scotland (2012–2017) found that the total count of registered outlets and the mean neighbourhood-level tobacco retail density decreased for all registered outlets following the introduction of point-of-sale legislation in supermarket (2013) and smaller shops (2015), with a sharp decline noted from 2013 (10, 206) to 2015 (8847) and remaining stable from 2016 (9042) to 2017 (9118) (Pearce et al., 2020). The current study suggests that, although there has been a marginal increase in the total count of ‘outlets selling tobacco’ (a closer measure than ‘all’ when making comparison with the earlier study as NVP registration wasn't introduced until 2017) from 8249 (2020) to 8293 (2024), the overall declining trend for all registered outlets that were observed in the previous study continued. We further identified a clear declining trend for tobacco-only outlets and a rise for outlets selling NVPs (i.e., outlets selling both tobacco and NVPs and NVP-only outlets). These findings may indicate there has been a pivot among retailers in what they are selling which likely reflects a more diverse market as consumers alter their consumption patterns. This pivot could be driven by multiple factors. First, NVPs are considered a smoking cessation tool in the UK (Department of Health and Social Care, 2023; Public Health England, 2026) though systematic reviews suggest the effectiveness of vaping as a cessation intervention has mixed results (Pound et al., 2021; Lindson et al., 2025). In the meantime, the prevalence of NNP use, which includes vapes and nicotine pouches, among both adults and youth has significantly increased (Brose et al., 2025; Public Health England, 2026; Jackson et al., 2024). Second, it may also point to the tobacco industry's strategic pivot, from traditional tobacco products to NNPs (Philip Morris International, 2026), which, for small retailers, are more profitable (Tunstall et al., 2024).

Consistent with what was observed in 2012–2017 (Pearce et al., 2020), the clear social gradient in neighbourhood-level retail density for all types of registered outlets, as well as the greater exposure in large urban areas, was also observed in the current analysis using data from 2020 to 2024. However, some nuanced discrepancies were noticed. In the previous study, the minor increase in the social gradient in 2015–2017 was mostly driven by the rising retailer density in the most deprived neighbourhoods (Pearce et al., 2020). In our study, the increase in the social gradient for all types of registered outlets in 2020–2024 was driven by both the increasing density in the most deprived neighbourhoods and the declining density in the least deprived neighbourhoods simultaneously. Given the more dramatic downward trend in density for tobacco-only outlets in more deprived neighbourhoods and the constant trend for outlets selling tobacco, it is likely that the overall rising social gradient was driven by the sharper increase in density for 'outlets selling tobacco and NVPs' in more deprived neighbourhoods. Notably, the spatial differences in neighbourhood-level density for NVP-only outlets are not as pronounced as those for other types of outlets, which may suggest that even though there has been a market shift from traditional tobacco to NNPs in Scotland, this shift is mostly driven by the outlets selling both tobacco and NVPs rather than NVP-only outlets, given the less clear pattern that was noticed for NVP-only outlets across urban-rural categories. Furthermore, it is possible that the growing density of NVP-only outlets was not fully driven by its more rapid increase in large urban areas, especially given that NVPs have been considered a replacement for smoking cessation.

The key strengths of our study are twofold. First, our study uses register data to provide longitudinal insights into changes in neighbourhood-level tobacco retail density at a national scale. Second, the results are stratified by the type of outlets (defined by the products sold) which provides insights into the shift in the local-level tobacco retail market, particularly for NVPs. There are limitations should be noted. First, this study utilised the Register of Tobacco and Nicotine Vapour Product Retailers in Scotland. Although the register has required registration for all outlets selling tobacco and/or NVPs since 2017, the system relies on self-reporting and is vulnerable to delayed or missing registration, incorrect information, or delayed updates. However, the field audits suggest the register is largely accurate. Second, the higher proportion of neighbourhoods with zero density of NVP-only outlets, particularly in rural areas, was mostly driven by the 10-min walking distance search radius chosen for calculating KDE. However, the 10-min walking distance radius is a widely adopted measure in previous studies and allows comparisons over time (Shortt et al., 2015; Pearce et al., 2020).

## 5 Conclusions

Greater tobacco retail availability may encourage initiation and discourage cessation by increasing the opportunities to purchase tobacco products, creating competitive local markets, and enhancing the visibility of tobacco products (Pearce et al., 2012). Lower levels of tobacco retail density/proximity have been found to be associated with lower tobacco use (Lee et al., 2021). It is likely that the link between local retail availability of NNPs and the use of NNPs shares similar mechanisms. Given the rising density and the widening social inequalities of outlets selling tobacco and NVPs, and the persistent and increasing social gradient in health in Scotland, stricter legislation to restrict the display and retail availability of NNPs is anticipated, especially after the passage of the Tobacco and Vape Act 2026. Earlier legislation, including the introduction of tobacco point-of-sale display ban between 2013 and 2015, and the Environmental Protection (Single-use Vapes) (Scotland) Regulations 2024, has not included provision to regulate the retail availability of tobacco and NNPs in Scotland. Our findings highlight the need for effective regulatory approaches and comprehensive policies aimed at balancing the regulation of the availability of tobacco and NNPs to prevent initiation among younger populations and supporting cessation efforts through the use of NNPs.

## CRediT authorship contribution statement

**Chunyu Zheng:** Writing – review & editing, Writing – original draft, Visualization, Validation, Methodology, Formal analysis, Data curation, Conceptualization. **Niamh Shortt:** Writing – review & editing, Writing – original draft, Validation, Project administration, Methodology, Funding acquisition, Data curation, Conceptualization. **Asiya Hamid:** Writing – review & editing, Writing – original draft, Conceptualization. **Andy MacGregor:** Writing – review & editing, Writing – original draft, Methodology, Funding acquisition, Conceptualization. **Amber-Jane Morgan:** Writing – review & editing, Writing – original draft, Data curation, Conceptualization. **Allison Ford:** Writing – review & editing, Writing – original draft, Project administration, Methodology, Funding acquisition, Data curation, Conceptualization. **Jamie Pearce:** Writing – review & editing, Writing – original draft, Validation, Project administration, Methodology, Funding acquisition, Data curation, Conceptualization.

## Funding

The study is supported by the Chief Scientist Office (CSO) of the Scottish Government (CSO Project Ref: HIPS/24/12) and through the SPECTRUM Consortium which is a UK Prevention Research Partnership grant (MR/S037519/1), funded by the British Heart Foundation, Cancer Research UK, Chief Scientist Office of the Scottish Government Health and Social Care Directorates, Engineering and Physical Sciences Research Council, Economic and Social Research Council, Health and Social Care Research and Development Division (Welsh Government), Medical Research Council, National Institute for Health Research, Natural Environment Research Council, Public Health Agency (Northern Ireland), The Health Foundation and Wellcome. The views expressed are those of the author and not necessarily those of the funders. No competing interest declared.

## Declaration of competing interest

The authors declare that they have no known competing financial interests or personal relationships that could have appeared to influence the work reported in this paper.

## Data Availability

Data will be made available on request.

## References

[bb0005] Al-Delaimy W.K., Sim F. (2021). Electronic cigarettes and public health: a policy brief. Int. J. Epidemiol..

[bb0010] Bhatnagar A., Whitsel L.P., Blaha M.J. (2019). New and emerging tobacco products and the nicotine endgame: the role of robust regulation and comprehensive tobacco control and prevention: a presidential advisory from the American Heart Association. Circulation.

[bb0015] Brose L., Bunce L., Cheeseman H. (2025). Prevalence of nicotine pouch use among youth and adults in Great Britain—analysis of cross-sectional, nationally representative surveys. Nicotine Tob. Res..

[bb0020] Caryl F., Shortt N.K., Pearce J., Reid G., Mitchell R. (2019). Socioeconomic inequalities in children’s exposure to tobacco retailing based on individual-level GPS data in Scotland. Tob. Control.

[bb0030] Clemens T., Dibben C., Pearce J., Shortt N.K. (2020). Neighbourhood tobacco supply and individual maternal smoking during pregnancy: a fixed-effects longitudinal analysis using routine data. Tob. Control.

[bb0035] Department of Health and Social Care (2023). Stopping the start: our new plan to create a smokefree generation. https://www.gov.uk/government/publications/stopping-the-start-our-new-plan-to-create-a-smokefree-generation/stopping-the-start-our-new-plan-to-create-a-smokefree-generation#supporting-people-to-quit-smoking.

[bb0040] Ford A., MacKintosh A.M., Moodie C., Kuipers M.A.G., Hastings G.B., Bauld L. (2020). Impact of a ban on the open display of tobacco products in retail outlets on never smoking youth in the UK: findings from a repeat cross-sectional survey before, during and after implementation. Tob. Control.

[bb0045] Golder S., Hartwell G., Barnett L.M., Nash S.G., Petticrew M., Glover R.E. (2025). Vaping and harm in young people: umbrella review. Tob. Control.

[bb0050] Hammond D., Reid J.L. (2025). Trends in vaping and nicotine product use among youth in Canada, England and the USA between 2017 and 2022: evidence to inform policy. Tob. Control.

[bb0055] He Y., Shang C., Huang J., Cheng K.-W., Chaloupka F.J. (2018). Global evidence on the effect of point-of-sale display bans on smoking prevalence. Tob. Control.

[bb0065] Iacobucci G. (2025). Vaping overtakes smoking in Britain for first time. BMJ.

[bb0070] Jackson S.E., Shahab L., Tattan-Birch H., Brown J. (2024). Vaping among adults in England who have never regularly smoked: a population-based study, 2016–24. Lancet Public Health.

[bb0075] Jackson S.E., Shahab L., Buss V. (2026). The changing face of nicotine use in England: age-specific annual trends, 2014 to 2024. Addiction.

[bb0080] Kaur J., Goel S., Shabil M. (2025). Health impacts of electronic nicotine delivery systems: an umbrella review of systematic reviews. BMJ Open.

[bb0085] Kong A.Y., Henriksen L. (2022). Retail endgame strategies: reduce tobacco availability and visibility and promote health equity. Tob. Control.

[bb0090] Kong A.Y., Lee J.G.L., Halvorson-Fried S.M. (2025). Neighbourhood inequities in the availability of retailers selling tobacco products: a systematic review. Tob. Control.

[bb0095] Kuipers M.A.G., Best C., Wilson M. (2020). Adolescents’ perceptions of tobacco accessibility and smoking norms and attitudes in response to the tobacco point-of-sale display ban in Scotland: results from the DISPLAY study. Tob. Control.

[bb0100] Lee J.G.L., Kong A.Y., Sewell K.B. (2021). Associations of tobacco retailer density and proximity with adult tobacco use behaviours and health outcomes: a meta-analysis. Tob. Control.

[bb0105] Lindson N., Livingstone-Banks J., Butler A.R. (2025). Electronic cigarettes for smoking cessation. Cochrane Database Syst. Rev..

[bb0110] MacGregor A., Shields J., Hamid A. (2025). ‘I’d rather have worse gums than worse lungs’: young people’s views of nicotine pouches in the UK. Addiction.

[bb0115] Moon G., Barnett R., Pearce J., Thompson L., Twigg L. (2018). The tobacco endgame: the neglected role of place and environment. Health Place.

[bb0120] OBE JK (2022).

[bb0125] Pearce J., Barnett R., Moon G. (2012). Sociospatial inequalities in health-related behaviours: pathways linking place and smoking. Prog. Hum. Geogr..

[bb0130] Pearce J., Rind E., Shortt N., Tisch C., Mitchell R. (2016). Tobacco retail environments and social inequalities in individual-level smoking and cessation among Scottish adults. Nicotine Tob. Res..

[bb0135] Pearce J., Cherrie M., Best C. (2020). How has the introduction of point-of-sale legislation affected the presence and visibility of tobacco retailing in Scotland? A longitudinal study. Tob. Control.

[bb0140] Philip Morris International (2026). PMI announces ambition to become a more than two-thirds majority smoke-free company by 2030. Accessed 6 January. https://www.pmi.com/our-progress/pmi-announces-ambition-to-become-a-more-than-two-thirds-majority-smoke-free-company-by-2030/.

[bib177] Pound C.M., Zhang J.Z., Kodua A.T., Sampson M. (2021). Smoking cessation in individuals who use vaping as compared with traditional nicotine replacement therapies: a systematic review and meta-analysis. BMJ Open.

[bb0145] Public Health England (2026). Vaping better than nicotine replacement therapy for stopping smoking, evidence suggests. Accessed 6 January. https://www.gov.uk/government/news/vaping-better-than-nicotine-replacement-therapy-for-stopping-smoking-evidence-suggests.

[bb0150] Shortt N.K., Tisch C., Pearce J. (2015). A cross-sectional analysis of the relationship between tobacco and alcohol outlet density and neighbourhood deprivation. BMC Public Health.

[bb0155] Shortt N.K., Tisch C., Pearce J., Richardson E.A., Mitchell R. (2016). The density of tobacco retailers in home and school environments and relationship with adolescent smoking behaviours in Scotland. Tob. Control.

[bb0160] The Lancet Global H (2024). Tobacco endgame is closer than we think. Lancet Glob. Health.

[bb0165] The Scottish Government (2025). Register of Retailers Selling Tobacco and Nicotine Vapour Products (e-cigs). Accessed 2 June. https://tobacco.epass.service.gov.scot/assets/files/docs/register-of-retailers-selling-tobacco-and-nicotine-vapour-products-2_accessible.pdf.

[bb0170] Tunstall H., Shortt N.K., Niedzwiedz C.L., Richardson E.A., Mitchell R.J., Pearce J.R. (2018). Tobacco outlet density and tobacco knowledge, beliefs, purchasing behaviours and price among adolescents in Scotland. Soc. Sci. Med..

[bb0175] Tunstall H., Valiente R., Shortt N., Pearce J. (2024). *Appendix 1: Tobacco and vape profits: Analysis of EPOS data from small retailers in the United Kingdom in 2019 and* 2022*.* Data report in Written evidence on the retail licensing and product registration powers in the Tobacco and Vapes Bill. https://bills.parliament.uk/publications/57593/documents/5573.

[bib176] Yan D., Wang Z., Laestadius L. (2023). A systematic review for the impacts of global approaches to regulating electronic nicotine products. J Glob Health.

