# Supplementary material for: Longitudinal analysis of geographical inequalities in retail density of tobacco and nicotine vapour products in Scotland, 2020–2024
Source: Prev Med Rep. 2026 Aug 25;70:103611. doi: 10.1016/j.pmedr.2026.103611 (PMC13560534; doi:10.1016/j.pmedr.2026.103611)
Supplement: Supplementary file 1 [file mmc1.docx]

**Contents**

[**Supplementary I. The distribution of zero-density neighbourhoods**	2](#_Toc238845529)

[**S Figure 1.** The proportion of zero-density neighbourhoods, 2020-2024, by product type	3](#_Toc238845530)

[**S Figure 2.** The proportion of zero-density neighbourhoods by income deprivation quintiles, 2020-2024, by product type	3](#_Toc238845531)

[**S Figure 3.** The proportion of zero-density neighbourhoods by urban-rural categories, 2020-2024, by product type	4](#_Toc238845532)

[**Supplementary II. Sensitivity test using count measure (count per 1,000 population per neighbourhood)**	5](#_Toc238845533)

[**S Figure 4.** Changes in neighbourhood-level density (count measure), 2020-2024, by product type	5](#_Toc238845534)

[**S Figure 5.** Changes in neighbourhood-level density (count measure) by income deprivation quintiles, 2020-2024, by product type	5](#_Toc238845535)

[**S Figure 6.** Changes in neighbourhood-level density (count measure) by urban-rural categories, 2020-2024, by product type	6](#_Toc238845536)

[**S Figure 7.** The proportion of zero-density neighbourhoods (count measure), 2020-2024, by product type	6](#_Toc238845537)

[**S Figure 8.** The proportion of zero-density neighbourhoods (count measure) by income deprivation quintiles, 2020-2024, by product type	7](#_Toc238845538)

[**S Figure 9.** The proportion of zero-density neighbourhoods (count measure) by urban-rural categories, 2020-2024, by product type	7](#_Toc238845539)

[**Supplementary III.** **The count of registered outlets per local authority**	8](#_Toc238845540)

[**S Table 1.** Total count of registered outlets per local authority	8](#_Toc238845541)

[**S Table 2.** Total count of tobacco-only outlets per local authority	9](#_Toc238845542)

[**S Table 3.** Total count of NVP-only outlets per local authority	10](#_Toc238845543)

[**S Table 4.** Total count of outlets selling tobacco and NVPs per local authority	11](#_Toc238845544)

[**S Table 5.** Total count of outlets selling NVPs per local authority	12](#_Toc238845545)

[**S Table 6.** Total count of outlets selling tobacco per local authority	13](#_Toc238845546)

# **Supplementary I. The distribution of zero-density neighbourhoods**

**Zero-density neighbourhoods’ distribution**

Given the excessive numbers of neighbourhoods with zero densities, particularly for NVP-only outlets, their distributions over time, by income deprivation quintiles and by urban-rural settings, have all been detailed in Supplementary I. In summary, aligning with the overall pattern observed for non-zero-density neighbourhoods, the proportion of neighbourhoods with zero densities was mostly constant over time for ‘all’ and ‘outlets selling tobacco’, where the proportion increased for ‘tobacco-only outlets’ and declined for ‘NVP-only outlets’, ‘outlets selling tobacco and NVPs’ and ‘outlets selling NVPs’. Additionally, it remained mostly constant over time that less deprived quintiles had a greater proportion of zero-density neighbourhoods, while the proportion declined in more rural areas for ‘outlet selling tobacco and NVPs’ and ‘outlets selling NVPs’.

However, we should note that ‘zero-density’ doesn’t necessarily mean that there are no outlets available to residents living in those neighbourhoods, but neighbourhoods with the lowest density, which reflects a lack of outlets within the search radius or the edge effect.

## **S Figure 1.** The proportion of zero-density neighbourhoods, 2020-2024, by product type

## **S Figure 2.** The proportion of zero-density neighbourhoods by income deprivation quintiles, 2020-2024, by product type

## **S Figure 3.** The proportion of zero-density neighbourhoods by urban-rural categories, 2020-2024, by product type

# **Supplementary II. Sensitivity test using count measure (count per 1,000 population per neighbourhood)**

## **S Figure 4.** Changes in neighbourhood-level density (count measure), 2020-2024, by product type

## **S Figure 5.** Changes in neighbourhood-level density (count measure) by income deprivation quintiles, 2020-2024, by product type

## **S Figure 6.** Changes in neighbourhood-level density (count measure) by urban-rural categories, 2020-2024, by product type

## **S Figure 7.** The proportion of zero-density neighbourhoods (count measure), 2020-2024, by product type

## **S Figure 8.** The proportion of zero-density neighbourhoods (count measure) by income deprivation quintiles, 2020-2024, by product type

## **S Figure 9.** The proportion of zero-density neighbourhoods (count measure) by urban-rural categories, 2020-2024, by product type

# **Supplementary III.** **The count of registered outlets per local authority**

## **S Table 1.** Total count of registered outlets per local authority

| **Council Area 2019 code** | **Council area** | **2020** | **2021** | **2022** | **2023** | **2024** |
| --- | --- | --- | --- | --- | --- | --- |
| S12000005 | Clackmannanshire | 82 | 83 | 75 | 79 | 78 |
| S12000006 | Dumfries and Galloway | 360 | 363 | 364 | 361 | 373 |
| S12000008 | East Ayrshire | 243 | 242 | 249 | 255 | 262 |
| S12000010 | East Lothian | 161 | 161 | 169 | 168 | 171 |
| S12000011 | East Renfrewshire | 115 | 117 | 120 | 105 | 108 |
| S12000013 | Na h-Eileanan Siar | 59 | 59 | 61 | 65 | 66 |
| S12000014 | Falkirk | 245 | 246 | 258 | 265 | 275 |
| S12000017 | Highland | 436 | 435 | 446 | 458 | 436 |
| S12000018 | Inverclyde | 128 | 127 | 129 | 130 | 129 |
| S12000019 | Midlothian | 167 | 171 | 173 | 179 | 183 |
| S12000020 | Moray | 163 | 166 | 173 | 181 | 163 |
| S12000021 | North Ayrshire | 269 | 266 | 270 | 285 | 252 |
| S12000023 | Orkney Islands | 68 | 68 | 70 | 70 | 72 |
| S12000026 | Scottish Borders | 172 | 175 | 177 | 191 | 197 |
| S12000027 | Shetland Islands | 73 | 69 | 64 | 60 | 59 |
| S12000028 | South Ayrshire | 202 | 192 | 191 | 182 | 188 |
| S12000029 | South Lanarkshire | 499 | 505 | 516 | 535 | 561 |
| S12000030 | Stirling | 176 | 174 | 150 | 151 | 154 |
| S12000033 | Aberdeen City | 339 | 340 | 329 | 297 | 326 |
| S12000034 | Aberdeenshire | 302 | 302 | 296 | 286 | 276 |
| S12000035 | Argyll and Bute | 231 | 234 | 227 | 225 | 223 |
| S12000036 | City of Edinburgh | 779 | 792 | 816 | 840 | 882 |
| S12000038 | Renfrewshire | 283 | 287 | 313 | 323 | 293 |
| S12000039 | West Dunbartonshire | 193 | 192 | 192 | 194 | 191 |
| S12000040 | West Lothian | 326 | 294 | 308 | 319 | 317 |
| S12000041 | Angus | 197 | 198 | 206 | 217 | 214 |
| S12000042 | Dundee City | 225 | 226 | 236 | 245 | 241 |
| S12000045 | East Dunbartonshire | 138 | 135 | 136 | 132 | 132 |
| S12000047 | Fife | 629 | 629 | 646 | 659 | 622 |
| S12000048 | Perth and Kinross | 224 | 226 | 229 | 238 | 247 |
| S12000049 | Glasgow City | 1416 | 1432 | 1509 | 1569 | 1635 |
| S12000050 | North Lanarkshire | 611 | 602 | 606 | 621 | 632 |

## **S Table 2.** Total count of tobacco-only outlets per local authority

| **Council Area 2019 code** | **Council area** | **2020** | **2021** | **2022** | **2023** | **2024** |
| --- | --- | --- | --- | --- | --- | --- |
| S12000005 | Clackmannanshire | 17 | 17 | 9 | 7 | 5 |
| S12000006 | Dumfries and Galloway | 188 | 188 | 183 | 146 | 141 |
| S12000008 | East Ayrshire | 98 | 97 | 94 | 90 | 86 |
| S12000010 | East Lothian | 89 | 89 | 87 | 76 | 71 |
| S12000011 | East Renfrewshire | 63 | 57 | 53 | 34 | 31 |
| S12000013 | Na h-Eileanan Siar | 42 | 42 | 43 | 44 | 44 |
| S12000014 | Falkirk | 57 | 55 | 51 | 50 | 50 |
| S12000017 | Highland | 151 | 152 | 152 | 150 | 111 |
| S12000018 | Inverclyde | 23 | 23 | 14 | 14 | 13 |
| S12000019 | Midlothian | 87 | 80 | 72 | 70 | 68 |
| S12000020 | Moray | 59 | 59 | 60 | 59 | 49 |
| S12000021 | North Ayrshire | 109 | 106 | 106 | 106 | 60 |
| S12000023 | Orkney Islands | 55 | 55 | 54 | 48 | 49 |
| S12000026 | Scottish Borders | 57 | 58 | 57 | 56 | 54 |
| S12000027 | Shetland Islands | 52 | 50 | 43 | 38 | 37 |
| S12000028 | South Ayrshire | 49 | 44 | 41 | 24 | 22 |
| S12000029 | South Lanarkshire | 104 | 105 | 101 | 88 | 88 |
| S12000030 | Stirling | 46 | 46 | 23 | 22 | 17 |
| S12000033 | Aberdeen City | 124 | 119 | 86 | 34 | 31 |
| S12000034 | Aberdeenshire | 82 | 77 | 69 | 57 | 43 |
| S12000035 | Argyll and Bute | 112 | 111 | 97 | 92 | 81 |
| S12000036 | City of Edinburgh | 387 | 387 | 384 | 357 | 356 |
| S12000038 | Renfrewshire | 82 | 84 | 80 | 71 | 46 |
| S12000039 | West Dunbartonshire | 92 | 91 | 87 | 80 | 67 |
| S12000040 | West Lothian | 165 | 111 | 98 | 88 | 65 |
| S12000041 | Angus | 74 | 76 | 74 | 75 | 70 |
| S12000042 | Dundee City | 22 | 19 | 16 | 16 | 13 |
| S12000045 | East Dunbartonshire | 57 | 56 | 54 | 44 | 35 |
| S12000047 | Fife | 225 | 223 | 202 | 179 | 128 |
| S12000048 | Perth and Kinross | 86 | 87 | 84 | 78 | 68 |
| S12000049 | Glasgow City | 844 | 839 | 799 | 761 | 755 |
| S12000050 | North Lanarkshire | 240 | 220 | 199 | 163 | 135 |

## **S Table 3.** Total count of NVP-only outlets per local authority

| **Council Area 2019 code** | **Council area** | **2020** | **2021** | **2022** | **2023** | **2024** |
| --- | --- | --- | --- | --- | --- | --- |
| S12000005 | Clackmannanshire | 19 | 19 | 19 | 22 | 23 |
| S12000006 | Dumfries and Galloway | 38 | 37 | 41 | 50 | 58 |
| S12000008 | East Ayrshire | 27 | 28 | 28 | 35 | 45 |
| S12000010 | East Lothian | 15 | 15 | 20 | 22 | 25 |
| S12000011 | East Renfrewshire | 10 | 10 | 11 | 11 | 13 |
| S12000013 | Na h-Eileanan Siar | 2 | 2 | 2 | 3 | 5 |
| S12000014 | Falkirk | 44 | 45 | 49 | 51 | 56 |
| S12000017 | Highland | 47 | 43 | 52 | 56 | 57 |
| S12000018 | Inverclyde | 26 | 25 | 26 | 27 | 23 |
| S12000019 | Midlothian | 22 | 21 | 23 | 30 | 31 |
| S12000020 | Moray | 26 | 27 | 31 | 35 | 27 |
| S12000021 | North Ayrshire | 31 | 30 | 30 | 42 | 42 |
| S12000023 | Orkney Islands | 1 | 1 | 3 | 3 | 4 |
| S12000026 | Scottish Borders | 26 | 26 | 25 | 31 | 34 |
| S12000027 | Shetland Islands | 2 | 2 | 1 | 1 | 2 |
| S12000028 | South Ayrshire | 37 | 32 | 30 | 31 | 33 |
| S12000029 | South Lanarkshire | 86 | 88 | 92 | 108 | 114 |
| S12000030 | Stirling | 26 | 25 | 21 | 23 | 27 |
| S12000033 | Aberdeen City | 43 | 43 | 47 | 53 | 67 |
| S12000034 | Aberdeenshire | 52 | 51 | 43 | 37 | 36 |
| S12000035 | Argyll and Bute | 27 | 29 | 34 | 30 | 32 |
| S12000036 | City of Edinburgh | 97 | 96 | 111 | 119 | 126 |
| S12000038 | Renfrewshire | 37 | 37 | 51 | 54 | 54 |
| S12000039 | West Dunbartonshire | 32 | 31 | 30 | 33 | 31 |
| S12000040 | West Lothian | 39 | 36 | 45 | 54 | 56 |
| S12000041 | Angus | 26 | 24 | 32 | 42 | 44 |
| S12000042 | Dundee City | 34 | 35 | 44 | 48 | 44 |
| S12000045 | East Dunbartonshire | 21 | 18 | 18 | 16 | 20 |
| S12000047 | Fife | 87 | 87 | 104 | 120 | 124 |
| S12000048 | Perth and Kinross | 26 | 24 | 26 | 35 | 45 |
| S12000049 | Glasgow City | 169 | 173 | 201 | 235 | 262 |
| S12000050 | North Lanarkshire | 87 | 87 | 87 | 91 | 105 |

## **S Table 4.** Total count of outlets selling tobacco and NVPs per local authority

| **Council Area 2019 code** | **Council area** | **2020** | **2021** | **2022** | **2023** | **2024** |
| --- | --- | --- | --- | --- | --- | --- |
| S12000005 | Clackmannanshire | 46 | 47 | 47 | 50 | 50 |
| S12000006 | Dumfries and Galloway | 134 | 138 | 140 | 165 | 174 |
| S12000008 | East Ayrshire | 118 | 117 | 127 | 130 | 131 |
| S12000010 | East Lothian | 57 | 57 | 62 | 70 | 75 |
| S12000011 | East Renfrewshire | 42 | 50 | 56 | 60 | 64 |
| S12000013 | Na h-Eileanan Siar | 15 | 15 | 16 | 18 | 17 |
| S12000014 | Falkirk | 144 | 146 | 158 | 164 | 169 |
| S12000017 | Highland | 238 | 240 | 242 | 252 | 268 |
| S12000018 | Inverclyde | 79 | 79 | 89 | 89 | 93 |
| S12000019 | Midlothian | 58 | 70 | 78 | 79 | 84 |
| S12000020 | Moray | 78 | 80 | 82 | 87 | 87 |
| S12000021 | North Ayrshire | 129 | 130 | 134 | 137 | 150 |
| S12000023 | Orkney Islands | 12 | 12 | 13 | 19 | 19 |
| S12000026 | Scottish Borders | 89 | 91 | 95 | 104 | 109 |
| S12000027 | Shetland Islands | 19 | 17 | 20 | 21 | 20 |
| S12000028 | South Ayrshire | 116 | 116 | 120 | 127 | 133 |
| S12000029 | South Lanarkshire | 309 | 312 | 323 | 339 | 359 |
| S12000030 | Stirling | 104 | 103 | 106 | 106 | 110 |
| S12000033 | Aberdeen City | 172 | 178 | 196 | 210 | 228 |
| S12000034 | Aberdeenshire | 168 | 174 | 184 | 192 | 197 |
| S12000035 | Argyll and Bute | 92 | 94 | 96 | 103 | 110 |
| S12000036 | City of Edinburgh | 295 | 309 | 321 | 364 | 400 |
| S12000038 | Renfrewshire | 164 | 166 | 182 | 198 | 193 |
| S12000039 | West Dunbartonshire | 69 | 70 | 75 | 81 | 93 |
| S12000040 | West Lothian | 122 | 147 | 165 | 177 | 196 |
| S12000041 | Angus | 97 | 98 | 100 | 100 | 100 |
| S12000042 | Dundee City | 169 | 172 | 176 | 181 | 184 |
| S12000045 | East Dunbartonshire | 60 | 61 | 64 | 72 | 77 |
| S12000047 | Fife | 317 | 319 | 340 | 360 | 370 |
| S12000048 | Perth and Kinross | 112 | 115 | 119 | 125 | 134 |
| S12000049 | Glasgow City | 403 | 420 | 509 | 573 | 618 |
| S12000050 | North Lanarkshire | 284 | 295 | 320 | 367 | 392 |

## **S Table 5.** Total count of outlets selling NVPs per local authority

| **Council Area 2019 code** | **Council area** | **2020** | **2021** | **2022** | **2023** | **2024** |
| --- | --- | --- | --- | --- | --- | --- |
| S12000005 | Clackmannanshire | 65 | 66 | 66 | 72 | 73 |
| S12000006 | Dumfries and Galloway | 172 | 175 | 181 | 215 | 232 |
| S12000008 | East Ayrshire | 145 | 145 | 155 | 165 | 176 |
| S12000010 | East Lothian | 72 | 72 | 82 | 92 | 100 |
| S12000011 | East Renfrewshire | 52 | 60 | 67 | 71 | 77 |
| S12000013 | Na h-Eileanan Siar | 17 | 17 | 18 | 21 | 22 |
| S12000014 | Falkirk | 188 | 191 | 207 | 215 | 225 |
| S12000017 | Highland | 285 | 283 | 294 | 308 | 325 |
| S12000018 | Inverclyde | 105 | 104 | 115 | 116 | 116 |
| S12000019 | Midlothian | 80 | 91 | 101 | 109 | 115 |
| S12000020 | Moray | 104 | 107 | 113 | 122 | 114 |
| S12000021 | North Ayrshire | 160 | 160 | 164 | 179 | 192 |
| S12000023 | Orkney Islands | 13 | 13 | 16 | 22 | 23 |
| S12000026 | Scottish Borders | 115 | 117 | 120 | 135 | 143 |
| S12000027 | Shetland Islands | 21 | 19 | 21 | 22 | 22 |
| S12000028 | South Ayrshire | 153 | 148 | 150 | 158 | 166 |
| S12000029 | South Lanarkshire | 395 | 400 | 415 | 447 | 473 |
| S12000030 | Stirling | 130 | 128 | 127 | 129 | 137 |
| S12000033 | Aberdeen City | 215 | 221 | 243 | 263 | 295 |
| S12000034 | Aberdeenshire | 220 | 225 | 227 | 229 | 233 |
| S12000035 | Argyll and Bute | 119 | 123 | 130 | 133 | 142 |
| S12000036 | City of Edinburgh | 392 | 405 | 432 | 483 | 526 |
| S12000038 | Renfrewshire | 201 | 203 | 233 | 252 | 247 |
| S12000039 | West Dunbartonshire | 101 | 101 | 105 | 114 | 124 |
| S12000040 | West Lothian | 161 | 183 | 210 | 231 | 252 |
| S12000041 | Angus | 123 | 122 | 132 | 142 | 144 |
| S12000042 | Dundee City | 203 | 207 | 220 | 229 | 228 |
| S12000045 | East Dunbartonshire | 81 | 79 | 82 | 88 | 97 |
| S12000047 | Fife | 404 | 406 | 444 | 480 | 494 |
| S12000048 | Perth and Kinross | 138 | 139 | 145 | 160 | 179 |
| S12000049 | Glasgow City | 572 | 593 | 710 | 808 | 880 |
| S12000050 | North Lanarkshire | 371 | 382 | 407 | 458 | 497 |

## **S Table 6.** Total count of outlets selling tobacco per local authority

| **Council Area 2019 code** | **Council area** | **2020** | **2021** | **2022** | **2023** | **2024** |
| --- | --- | --- | --- | --- | --- | --- |
| S12000005 | Clackmannanshire | 63 | 64 | 56 | 57 | 55 |
| S12000006 | Dumfries and Galloway | 322 | 326 | 323 | 311 | 315 |
| S12000008 | East Ayrshire | 216 | 214 | 221 | 220 | 217 |
| S12000010 | East Lothian | 146 | 146 | 149 | 146 | 146 |
| S12000011 | East Renfrewshire | 105 | 107 | 109 | 94 | 95 |
| S12000013 | Na h-Eileanan Siar | 57 | 57 | 59 | 62 | 61 |
| S12000014 | Falkirk | 201 | 201 | 209 | 214 | 219 |
| S12000017 | Highland | 389 | 392 | 394 | 402 | 379 |
| S12000018 | Inverclyde | 102 | 102 | 103 | 103 | 106 |
| S12000019 | Midlothian | 145 | 150 | 150 | 149 | 152 |
| S12000020 | Moray | 137 | 139 | 142 | 146 | 136 |
| S12000021 | North Ayrshire | 238 | 236 | 240 | 243 | 210 |
| S12000023 | Orkney Islands | 67 | 67 | 67 | 67 | 68 |
| S12000026 | Scottish Borders | 146 | 149 | 152 | 160 | 163 |
| S12000027 | Shetland Islands | 71 | 67 | 63 | 59 | 57 |
| S12000028 | South Ayrshire | 165 | 160 | 161 | 151 | 155 |
| S12000029 | South Lanarkshire | 413 | 417 | 424 | 427 | 447 |
| S12000030 | Stirling | 150 | 149 | 129 | 128 | 127 |
| S12000033 | Aberdeen City | 296 | 297 | 282 | 244 | 259 |
| S12000034 | Aberdeenshire | 250 | 251 | 253 | 249 | 240 |
| S12000035 | Argyll and Bute | 204 | 205 | 193 | 195 | 191 |
| S12000036 | City of Edinburgh | 682 | 696 | 705 | 721 | 756 |
| S12000038 | Renfrewshire | 246 | 250 | 262 | 269 | 239 |
| S12000039 | West Dunbartonshire | 161 | 161 | 162 | 161 | 160 |
| S12000040 | West Lothian | 287 | 258 | 263 | 265 | 261 |
| S12000041 | Angus | 171 | 174 | 174 | 175 | 170 |
| S12000042 | Dundee City | 191 | 191 | 192 | 197 | 197 |
| S12000045 | East Dunbartonshire | 117 | 117 | 118 | 116 | 112 |
| S12000047 | Fife | 542 | 542 | 542 | 539 | 498 |
| S12000048 | Perth and Kinross | 63 | 202 | 203 | 203 | 202 |
| S12000049 | Glasgow City | 322 | 1259 | 1308 | 1334 | 1373 |
| S12000050 | North Lanarkshire | 216 | 515 | 519 | 530 | 527 |
